# Multifunctional Polymer Composites Produced by Melt-Blown Technique to Use in Filtering Respiratory Protective Devices

**DOI:** 10.3390/ma13030712

**Published:** 2020-02-05

**Authors:** Agnieszka Brochocka, Aleksandra Nowak, Katarzyna Majchrzycka, Michał Puchalski, Sławomir Sztajnowski

**Affiliations:** 1 Department of Personal Protective Equipment, Central Institute for Labour Protection—National Research Institute, 90-133 Lodz, Poland; alnow@ciop.lodz.pl (A.N.); kamaj@ciop.lodz.pl (K.M.); 2 Faculty of Materials Technologies and Textiles Design, Lodz University of Technology,90-924 Lodz, Poland; michal.puchalski@p.lodz.pl (M.P.); slawomir.sztajnowski@p.lodz.pl (S.S.)

**Keywords:** polymer composites, biocide, superabsorbent polymer, filtering nonwoven, melt-blowing technique

## Abstract

In this work, a multifunctional polymer composite is made using melt-blowing technology from polypropylene (88 wt.%) and poly (ethylene terephthalate) (12 wt.%) with the addition of functional modifiers, that is, 3 g of a superabsorbent polymer and 5 g of a biocidal agent (Biohaloysite). The use of modifiers is aimed at obtaining adequate comfort when using the target respiratory protection equipment (RPE) in terms of microclimate in the breathing zone and protection against harmful aerosols including bioaerosols. The developed production method is innovative in that the two powdered modifiers are simultaneously applied in the stream of elementary polymeric fibers by two independent injection systems. Aerosols of the modifiers are supplied via a specially designed channel in the central segment of the die assembly, reducing the amount of materials used in the production process and saving energy. The results show that the proposed method of incorporating additives into the fiber structure did not adversely affect the protective and functional properties of the resulting filtration nonwovens. The produced nonwoven composites are characterized by SEM, FTIR, and differential scanning calorimetry (DSC). Given their high filtration efficiency at 5%, satisfactory airflow resistance (~200 Pa), very good antimicrobial activity, and excellent water absorption capacity, the obtained multifunctional nonwoven composites may be successfully used in filtering respiratory protective devices.

## 1. Introduction

Filtering respiratory protective devices (FRPDs) are used in situations involving an immediate threat to human health or life. They provide protection against hazards that may be difficult to predict or observe by workers. Thus, it is critical to intensify efforts to develop effective FRPDs which ensure the highest possible user comfort. Nonwovens applied in FRPDs should meet requirements concerning effective filtration of harmful aerosols and a low pressure drop at the filter, as well as some specific criteria associated with the intended use of the equipment. Of particular importance is user comfort, which is determined by the microclimate in the breathing zone. In FRPDs relative air humidity under the facepiece is always elevated, reaching up to 100%. During use, water vapor particles condense on the inner side of the device, leading to a dramatic deterioration of the microclimate. This adverse phenomenon becomes more acute during strenuous work, when minute ventilation increases while aerosol and water vapor particles form a so-called particle cake, rapidly leading to a rise in air pressure and breathing resistance. At the same time, these adverse conditions are conducive to the development of microorganisms that accumulate in the nonwoven during respiration.

This is especially true where FRPDs are used for protection against biological hazards (harmful aerosols), e.g., in health care, mining and geology, the cosmetic and pharmaceutical industries, and biotechnology. FRPDs (half-masks/facepiece respirators and filters) are made from two or three nonwoven layers, each characterized by some specific functional requirements. Those layers are usually made using different technologies and exhibit different porosities. As a consequence, the final products are rather thick, which makes them difficult to fit to the user’s face and unfavorably increases temperature and relative humidity inside the facepiece.

Recent years have seen considerable growth in the manufacture of melt-blown nonwoven composites modified to obtain specific properties. Melt-blown nonwovens are characterized by small-diameter fibers (less than 1 μm) which offer excellent filtration properties, thermal insulation, and sorption capacity [1]. Melt-blowing technology can be used to produce both hydrophilic and hydrophobic materials and also incorporate additives to improve filtration efficiency, moisture adsorption, and biocidal properties. Literature data [2] indicate that fiber diameter, pore size, and areal density have a great impact on the performance of the filter nonwoven in terms of protective and functional parameters. Many modification techniques have been described in the literature to date. In the study described in paper [3], the authors developed a new method of supplying modifiers to polymeric fibers: an electrostatic charge was applied to the fibers and a polymeric superabsorbent (SAP) was fed directly to the fiber-forming area in the melt-blowing process. The resulting nonwovens included outer-layer composites containing SAP with varying grain sizes as well as filtering nonwovens with and without electrostatic charges. The developed materials were characterized by high filtration performance and air moisture sorption capacity at good air flow [1]. Another study examined the effects of ε-caprolactone on the structure, morphology, mechanical properties, and filtration efficiency of a poly (lactic acid)/poly (ε-caprolactone) melt-blown nonwoven. Tributyl citrate (3 wt.%) was added to poly (ε-caprolactone) as a composite compatibilizer. The addition of poly (ε-caprolactone) was found to increase the fiber diameter and decrease the filtration efficiency of the resulting nonwoven [4]. Okrasa et al. added nanometer- and micrometer-scale octavinyl-POSS (polyhedral silsesquioxanes) particles to a polymeric matrix, which doubled the electrostatic potential of the obtained filtration materials. The nanofiller also led to smaller elementary fiber diameters as compared to unmodified fibers [5]. Those results were corroborated by Xiaoyan Song et al., who improved mechanical, electrostatic, and filtration properties by incorporating a POSS nanofiller into a polypropylene melt [6]. The addition of magnesium stearate (MgSt) to the polypropylene melt in a melt-blown process increased its electrostatic potential and improved its filtration properties and air permeability (99.22% filtration efficiency with an airflow resistance of 85 L/min at 92 Pa). The developed composite was characterized by excellent electrostatic stability ensuring long storage and use times [7].

Considerable research efforts have been made to obtain filtering nonwoven composites with antimicrobial properties. An interesting example is a polylactyd (PLA)/polypropylene (PP)/paraffin/CuO⋅SiO_2_ (47.25/47.25/5/0.5%, wt.%) composite which was obtained in a one-step melt-blowing process. It revealed strong antibacterial properties, slight activity against yeast, and improved filtration efficiency [8]. The study conducted by Majchrzycka et al. focused on developing high-performance melt-blown nonwovens containing gemini surfactants with time-dependent biocidal activity. Antimicrobial properties were tested under conditions simulating work at a plant biomass processing unit. The obtained nonwovens were found to exhibit high levels of protection against aerosol (0.7% penetration at an air flow resistance of 300 Pa) and varied levels of antimicrobial activity. Higher antimicrobial activity was noted for bacteria (max R = 87.85–97.46%) and lower for molds (max R = 80.11–94.53%). The developed PP/SPBS (a set of porous biocidal structures) filtering nonwovens with biocidal activity were deemed suitable for the production of re-usable FRPDs [9].

Brochocka conducted technological studies aimed at improving electret melt-blown polycarbonate (PC) nonwovens using modifiers with different electrostatic potentials to produce filtering materials which could effectively stop nanoparticles. The modifiers (perlite and amber granules) were supplied directly into the fiber-forming zone of the die assembly (where the extruded thermoplastic polymer is air-blown into fine fibers), due to which the modifier particles were strongly bonded to the microfibers. Brochocka’s method of producing electret melt-blown PC nonwovens was found to substantially increase the filtration performance [10]. In turn, paper [11] has presented the innovative technique of introducing liquid modifiers into filtration melt-blown nonwovens involving a special device implemented in the channel of the die assembly to supply rosin solutions directly to the polymer stream in the fiber-forming zone (affecting fiber thickness). The filtration properties of modified PP and PC nonwovens were tested against nanoaerosols depending on the concentration of rosin solutions. The obtained filtration material, which was not electrostatically activated, exhibited high initial penetration by nanoaerosols.

Commercially available FRPDs do not contain layers that exhibit both effective moisture-absorbing and bioactive properties. On the other hand, some authors have reported FRPDs made from polypropylene [12] as well as needle-punched nonwovens incorporating superabsorbent fibers [13] (in the latter case the focus has been on the sorption of liquids by nonwovens intended for sweat-absorbing inserts).

The objective of the present work was to develop a melt-blowing technology that would enable the simultaneous incorporation of two modifiers (a solid superabsorbent and a biocidal agent) to obtain polymeric composites for filtering protective respiratory devices. The filtration, functional, and biocidal properties of the resulting nonwovens are determined. The innovative feature of the proposed solution is the existence of nonwoven production and modification within one technological process.

## 2. Materials and Methods

### 2.1. Materials—Polymers and Additivies

A multifunctional polymeric composite was made from Borealis Borflow HL 508 FB PP granulate with a melt-flow index (MFI) of 800 g/10 min from Secura B.C. (Warsaw, Poland) and Skypet poly (ethylene terephthalate) (PET) from S.I. Zgoda (Konstatynow Lodzki, Poland). The biocidal additive was Biohaloysite from MDA Sp. z o.o. (Poznan, Poland) and the polymeric superabsorbent was fine-powdered EK-X EN 52 from Nippon/Ecotec (Torun, Poland). PET and PP were mixed at a ratio of 12:88 (wt.%), melted in the extruder barrel, and thoroughly homogenized. Moreover, 3 g of fine SAP powder per square meter and 5 g of Biohaloysite per square meter were directly supplied into the fiber-forming head. Biohaloysite in the form of didecyldimethylammonium chloride (CAS 7173-51-5, WE 230-525-2) was used as the active substance with bactericidal properties, and has been approved for use as an active substance with antimicrobial effects in accordance with the Regulation of the European Parliament and of the Council (EU) No. 528/2012 of 22 May 2012 regarding the making available on the market and use of biocidal products. This agent was embedded in halloysite nanotubes in the presence of substances that increase their wettability. SAP polymer with grain sizes of 30 μm was selected from the group of polymers or copolymers of acrylic acid or their sodium salts. The proportions of modifiers and polymers used were selected through a series of experimental works. Based on them, the variant characterized by the most optimal and satisfactory values of protective and operational parameters was selected.

The use of a mixture of PP with a PET-doped component enhanced the effect of the electrostatic attraction of particles from polluted air. An assessment of the filtration properties of the filtering nonwovens was carried out by testing the penetration into the mist of paraffin oil mist (Table 1).

Based on the results obtained, it was observed that the introduction of PET polymer in an amount of 12 wt.% to polypropylene helped to improve the filtration properties of the nonwoven material. The average penetration value for the paraffin oil mist improved by 66% compared to the PP-based nonwoven. The variants produced were subjected to thermal conditioning (T.C.), which consisted of their storage for 24 h at 70 ± 3 °C, and then 24 h at −30 ± 3 °C. There was a 4 h time interval between cycles that allowed samples to return to room temperature. T.C. caused a decrease in the filtering properties of nonwovens. For WP_0_ nonwoven, the penetration value increased by 51% compared to the nonwoven before T.C. The increase in penetration was due to the loss of an electrostatic charge applied during the nonwoven fabric production process. The penetration of paraffin oil mist for the variant reference filtration nonwoven (WSB_0_) increased by only 13.5%. In connection with the above, the WSB_0_ nonwoven variant containing PET-doped PP polymer was selected for further research.

The produced filtration nonwoven composite (designated WSB_1_) and the reference filtration nonwoven WSB_0_ were characterized, with the results shown in Table 2.

The nonwoven with the addition of SAP and Biohaloysite (WSB_1_) was thicker and had greater surface density, by 45% and 80%, respectively, compared to the reference nonwoven. This is attributable to the fact that the additives incorporated into WSB_1_ were characterized by small grain size.

### 2.2. Processing Equipment

The technological line for the production of melt-blown nonwovens consisted of

- an extruder
- a die assembly
- an air heater
- an electrostatic activation device, and
- a collector.

In addition, the production setup was equipped with inverters, refrigeration dryers, constant-voltage regulators, autotransformers, voltage meters, and sensors measuring the temperature of the bottom part of the die assembly. The basic technological parameters of the melt-blown process are shown in Table 3 and refer to the best variant.

**Figure 1:**
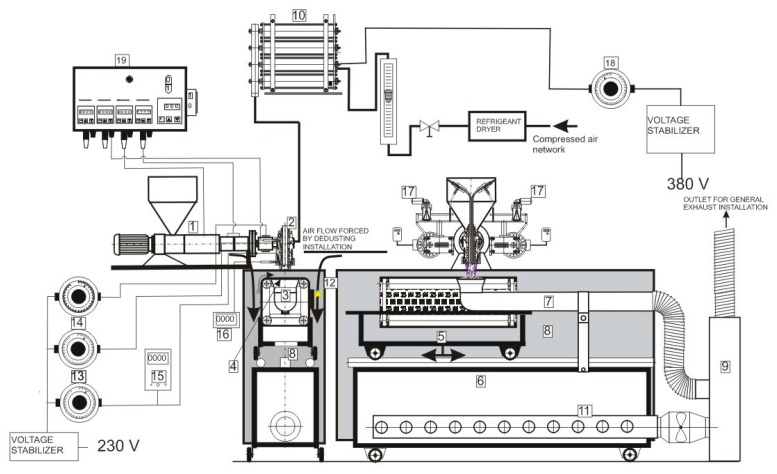
Diagram of the melt-blowing setup for the fabrication of multifunctional polymeric composites. 1—polymer extruder, 2—fiber-forming head, 3—fiber-attracting nozzle, 4—fiber-embedding mesh, 5—carriage shifting the mesh perpendicular to the extruder axis, 6—trolley enabling the device to exit from under the fiber-forming head (for cutting off the sheet), 7—vacuum installation, 8—enclosure walls, 9—air filter with modifiers, 10—air heater, 11—perforated pipe for dust extraction, 12—electrostatic activator, 13—autotransformer supplying the lower head heater, 14—autotransformer supplying the upper head heater, 15—voltmeter, 16—polymer melt temperature indicator, 17—set of devices for introducing modifiers, 18—autotransformer supplying air heaters, 19—extruder control cabinet.

The production of multifunctional polymer composites involves air-blowing of the polymer melt into elementary fibers of various thicknesses and lengths. PP granules are fed from the hopper to the extruder barrel, where the polymer is plasticized and homogenized. The temperatures of the three extruder zones affect the density of the polymer melt, which is critical in polymer flow rate and obtaining fibers with appropriate diameters (<1 μm). The amount of extruded melt is regulated by screw rotation. The compressed air supplied to the air heater is dried and heated to the desired temperature. Subsequently, the air stream is directed to the die assembly, where the polymer melt is heated to the final temperature. The melt extruded from the nozzles is attenuated by hot air jets into elementary fibers. The polymeric fibers with embedded modifiers are deposited on the collector screen that is being rotated by four rollers over an immobile suction box. In this work, the experimental setup was equipped with several checkpoints for the measurement of technological parameters and process control. A diagram of the setup is given in Figure 1.

**Figure 2:**
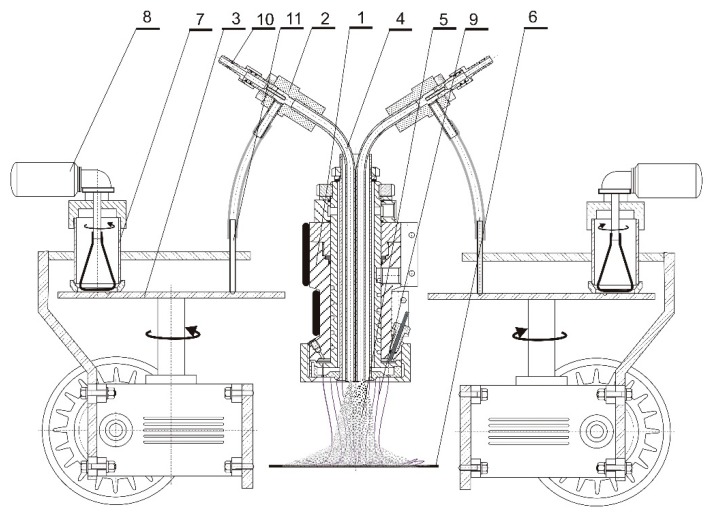
The process of applying two modifiers to the stream of elementary fibers in melt-blown technology. 1—fiber-forming head, 2—injector, 3—modifier dispenser plate, 4—tube transporting the modifiers to the fiber-forming zone, 5—mixed modifiers, 6—nonwoven layer, 7—modifier tank, 8—stirrer drive, 9—polymeric monofilaments, 10—compressed air inlet, 11—modifier intake suction nozzle.

The proposed die assembly is compatible with any thermoplastic polymer and can be readily implemented with devices feeding various forms of modifiers [14]. In the present study, powdered modifiers were supplied via an injector system. In such a setup, several injection modules can be installed to simultaneously feed two or more modifiers to be incorporated into the structure of filtration nonwovens at the stage of forming fibers from plasticized polymer. In this way, modifier particles become well embedded in the fibers immediately after polymer extrusion from the die. Furthermore, the incorporated modifiers remain active for an expected period of time across the nonwoven. The pneumatic application of powdered modifiers eliminates the need to fabricate multiple nonwoven layers, containing one modifier each, to be subsequently bonded together into a composite. A diagram showing the proposed method for the simultaneous application of two modifiers in the stream of elementary fibers is given in Figure 2.

The novelty of the solution consists of the use of two pneumatic devices to apply modifiers. Injectors powered by compressed air were connected to the top part of the die assembly. The compressed air flowing through the injector nozzle created a vacuum in the aerosol mixing vessel and sucked in the modifier from the dispenser plate. Diluted by the expanding air, the modifier aerosol was supplied to the fiber-forming zone via a tube placed in the channel of the die assembly. The presented method may be used for the application of powdered modifiers characterized by low thermal stability as the proposed construction of the die assembly considerably shortens their dwell time in the heated zone. This solution offers a convenient way to achieve symmetrical application of two additives within the center of the fiber-forming zone. As a result, the additives become simultaneously embedded in the structure of the fabricated nonwoven.

### 2.3. Testing Methods

The produced multifunctional polymeric composites were tested in a laboratory to determine the following parameters: filtration efficiency in terms of paraffin oil mist penetration (0.4 μm particle diameter), functional properties (airflow resistance at a volumetric flow rate not greater than 340 L/min), and biocidal activity. Moreover, the study examined the effects of the incorporated additives on the water absorption capacity of the nonwoven. Additionally, the morphological structures of materials were investigated by the use of scanning electron microscopy (FEI, Eindhoven, The Netherland), and IR spectroscopic analysis (Thermo Scientific, Waltham, MA, USA) was achieved by means of an FTIR spectrometer. Thermal analysis of produced composite nonwovens was performed using differential scanning calorimetry (DSC, TA Instruments, Royal Manor Crawley, West Sussex UK).

#### 2.3.1. Scanning Electron Microscopy

The morphological structures of the multifunctional composite materials were studied by using the high-resolution scanning electron microscope Nova NanoSEM 230 (SEM; FEI, Eindhoven, The Netherlands). The investigations were carried out under low vacuum conditions (0.7 mbar) with electron beam energy 10 keV. Test samples were prepared by mounting them to pin stubs using carbon conductive adhesive tape.

#### 2.3.2. Fourier Transform Infrared Spectroscopy

IR spectroscopic analysis was carried out using the reflectance technique with the use of an FTIR spectrophotometer, namely, the Nicolet 6700 (Thermo Scientific, Waltham, MA, USA), and a Smart iTR with Dia crystal with a 45° reflection angle. FTIR-ATR (Attenuated Total Reflectance) absorption spectra were recorded within a wavelength range of 4000 cm^−1^ and 600 cm^−1^ in the system A = f (1/λ). The OMNIC 0.8 program was used to analyze the obtained spectrograms and determine characteristic bands in the 700–3800 cm^−1^ range.

#### 2.3.3. Differential Scanning Calorimetry

The thermal characteristics of the studied materials, such as the cold crystallization temperature (T_cc_) and the melting temperature (T_m_), were determined using a differential scanning calorimetry Q2000 device (TA Instruments, Royal Manor Crawley, West Sussex UK) that was calibrated with indium. All measurements were made at a heating rate of 10 °C min^−1^ over a temperature range of −50–300 °C in a dry nitrogen environment according to the PN-EN ISO 11357:2009 Standard [15]. Additionally, the enthalpy of crystallization upon heating (ΔH_cc_) and of melting (ΔH_m_) were estimated.

#### 2.3.4. Water Absorption Capacity

Water absorption capacity was measured by means of the gravimetric method. Distilled water was used to eliminate the effects of salt and other impurities. Composite samples with a diameter of 80 mm were individually weighed, placed in beakers, and immersed in 200 mL of distilled water. After 20 min, the samples were drained on filter paper, transferred to previously weighed Petri dishes, and weighed again. Water absorption capacity *P* was calculated using the equation

(1) $$P\;=\;\frac{w_{1}-\;w_{2}}{w_{1}}$$

where *w*_1_ and *w*_2_ stand for the weight of the sample before and after the test, respectively. Eight independent replicates were made for each composite variant and the reference nonwoven.

#### 2.3.5. Antimicrobial Activity—the Dynamic Method

The antimicrobial activity of the multifunctional polymeric composite was evaluated against bacteria from the LOCK 105 Pure Culture Collection (see Table 4). The viability of those microorganisms was tested using an experimental stand at the laboratory of the Institute of Fermentation Technology and Microbiology, Lodz University of Technology [16].

The applied bioaerosol was characterized with a mean particle diameter of 0.5 μm. Tests were conducted in a pneumatic chamber with a HEPA filter (High Efficiency Particulate Air filter) and a UV lamp, in which the samples were placed. The bioaerosol was generated in an atomizer and mixed with a stream of dry air which was passed through the tested polymeric composite filter followed by a microbiological filter at a laminar air flow of 30 L/min. The microbiological filter was used to evaluate the proportion of bacteria stopped by the upstream tested filter. The filters were exposed to the generated aerosol for 20 min.

#### 2.3.6. Paraffin Oil Mist Penetration

Paraffin oil mist penetration tests were conducted using a Lorenz AGW-F/BIA generator (LORENTZ, Lindau, Germany), a Lorenz AP2E laser photometer (LORENTZ, Lindau, Germany), and an FH 143/149 pneumatic sample holder (LORENTZ, Lindau, Germany) (100 mm in diameter). Paraffin oil mist from the aerosol generator was passed through composite samples placed in the pneumatic holder at a linear speed of 95 L/min. Aerosol concentration upstream and downstream of the sample was measured using a Lorenz AP2E laser photometer. The distribution of paraffin oil mist particles was log-normal with a median Stokes diameter of 0.4 μm at a geometric standard deviation of 1.82. Paraffin oil mist penetration *P_POM_* was calculated using the formula

(2) $$P_{POM}\;=\;\frac{l_{2}-\;l_{0}}{l_{1}-\;l_{0}}\;\times 100\%$$

where *l*_0_, *l*_1_, and *l*_2_ are photometric readings for pure air (*l*_0_) and paraffin oil mist upstream (*l*_1_) and downstream (*l*_2_) of the sample. Readings were made after 3 min in the initial stage of filtration. Paraffin oil mist penetration was determined in accordance with the methodology stipulated in the European standards concerning requirements and testing methods for filtering half-masks EN 149:2001+A1:2009 [17] and EN 13274-7:2008 [18].

#### 2.3.7. Airflow Resistance

Airflow resistance is a basic parameter determining the functional properties of filtering respiratory devices. It was measured together with paraffin oil mist penetration. The results were read using a CMR-10A digital micromanometer measuring the gas pressure differential upstream/downstream of the filtering medium. The test was conducted in accordance with the requirements of EN 149:2001+A1:2009 [17] and EN 13274-3:2001 [19].

## 3. Results

### 3.1. SEM Results

**Figure 3:**
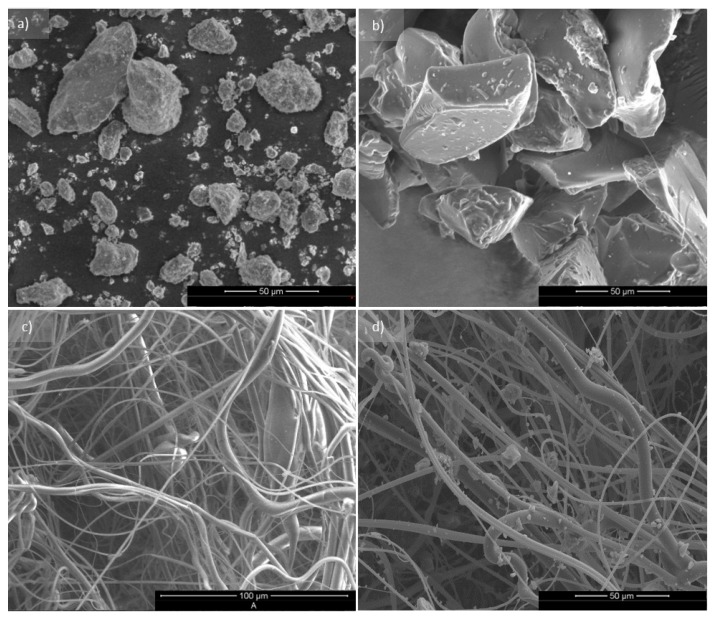
SEM images of Biohaloysite. (**a**) Polymeric superabsorbent (SAP), (**b**) unmodificated nonwoven WSB_0_, (**c**) nonwoven with functional additives WSB_1_, and (**d**) at a magnification of 1600×.

In Figure 3 a comparison of the unmodified nonwoven and the composite with SAP and Biohaloysite is presented. The fibrous structures of both materials are similar in the frame of fiber orientation and fiber diameter and the only difference is the presence of homogeneously redistributed additives. Large grains of superabsorbent polymer are permanently incorporated into the nonwoven structure. On the other hand, much smaller particles of Biohaloysite adhere to the fibers, which should have been expected. SEM research allowed us to visualize the structures and demonstrate their homogeneity, which was the aim of the developed method of the creation of functional composite materials based on melt-blow nonwovens.

**Figure 4:**
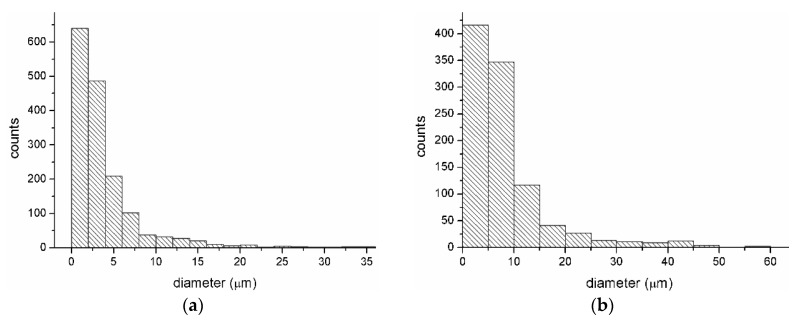
Particle size of (**a**) Biohaloysite and (**b**) SAP.

**Figure 5:**
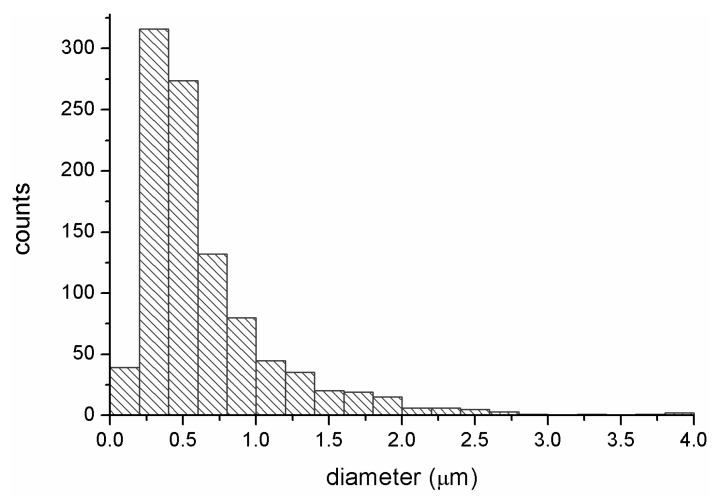
Particle size histogram of fibers in nonwoven WSB_1_.

Graphical interpretations regarding particle size of biocide, superabsorbent polymer, and elementary fiber size are shown below (Figure 4 and Figure 5). For this purpose we used OrginLab Pro 8.6 software. The graphs were created as two-dimensional planes where one axis corresponds to the results of the number of particles or fibers and the other represents the size of their sizes.

On the base of the presented results (Figure 4a) it was found that the particles are characterized by sizes from 0.0–2.5 µm and occur in the largest quantity (around 700) for both populations. Large quantity participation is also shown for biocide particles 2.5–4.0 μm in size, and particles in the size range 5.0–25.0 μm make up around 26% of the total collection. The particle size distribution of the SAP modifier is shown in Figure 4b. For that based on obtained data it was found that 78% of the total population are particles in the size range 0.0–10.0 μm, and that only 10% of particles are in the size range 10.0–15.0 μm.

The dimensions of elementary fiber diameters, which constitute the structure of the filtering nonwovens (WSB_1_), were also analyzed. On their basis (Figure 5), it was observed that the largest percentage (over 60%) was made up of fibers with a diameter in the range 0.25–0.70 μm. In addition, fibers with a diameter of 0.6–0.8 μm were also identified, and have a population of around 130 against the background of the total population.

### 3.2. Fourier Transform Infrared Spectroscopy

**Figure 6:**
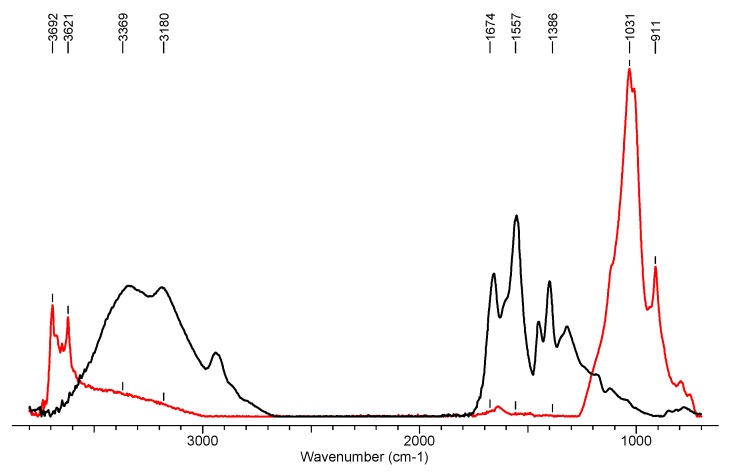
FTIR spectrum for modifiers: SAP (1—Black line) and Biohaloysite (2—Red line).

**Figure 7:**
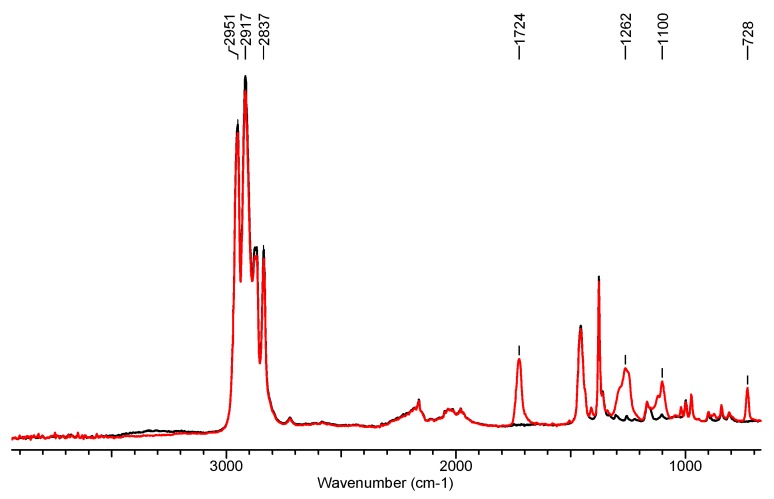
FTIR spectrum of nonwoven based on polypropylene WP_0_ (1—black line) and nonwoven WSB_0_ (2—red line).

The aim of IR absorption spectroscopy was to determine the differences in the chemical structure of the modified samples, namely, nonwoven of polypropylene (WP_0_), polypropylene/poly (ethylene terephthalate) (WSB_0_), and polypropylene/poly (ethylene terephthalate) with modifiers (WSB_1_). Changes in the molecular structure of the surface layer of the nonwovens were determined. Spectrograms characterizing the chemical structure of the tested samples are presented below (Figure 6 and Figure 7).

Figure 7 shows the IR spectra for nonwoven PP (WP_0_) and nonwoven PP-doped PET (WSB_0_). When analyzing infrared spectra, the emergence of a new band of about 1725 cm^−1^ was observed for the variant WSB_0_. The presence of this signal for spectrum 2 indicates the presence of a carbonyl group (C=O) derived from the group of polyesters. Its presence allows us to state that in the structure of the analyzed nonwoven fabric there is additionally a thermoplastic PET polymer. The combination of spectra allowed the identification of the chemical composition of individual nonwovens. In addition, the tests confirmed the use of a blend of PP and PET polymers in the target nonwoven fabric for use in protection against harmful bioaerosol particles.

### 3.3. Differential Scanning Calorimetry

**Figure 8:**
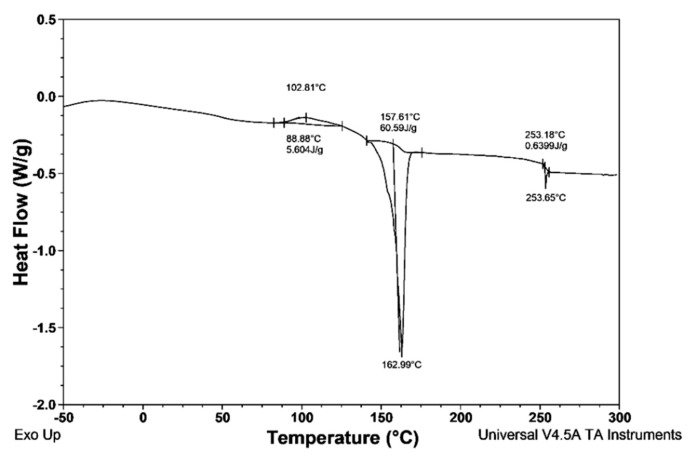
Thermograph of nonwoven with functional additives WSB_1_.

The thermal characteristics of the preformed modified nonwoven were analyzed by the use of differential scanning calorimetry. In Figure 8, a DSC thermograph with characteristic temperatures and calculated enthalpies of the studied material is presented.

The first peak is an exothermic peak with a maximum at 102 °C which corresponds to cold crystallization of PP, which dominated the polymer in the studied material composition. The next clearly visible peak is an endothermic peak with onset and maximum values at 157 °C and 163 °C, respectively. The enthalpy of this peak is 60.59 J/g, which corresponds to a crystallinity of around 28%, assuming ΔH100% equals 207 J/g [20]. The low degree of crystallinity testifies that the addition of powdered materials such as SAP and Biohaloysite do not result in significant crystallization in the melt-blow processing of PP. The DSC thermograph contains also a small peak at 253 °C, which, in our opinion, corresponds to the melting point of a small amount of low-crystalline PET.

### 3.4. Water Absorption Capacity

Water absorption capacity results for the produced multifunctional polymeric nonwoven containing 3 g of SAP and 5 g of the biocidal agent Biohaloysite (WSB_1_), and for the reference nonwoven (WSB_0_), are presented in Table 5.

The results showed that the water absorption capacity of WSB_1_ was higher than that of WSB_0_ by approximately 40%. The high standard deviation values may be attributable to the non-homogeneous distribution of modifiers in the structure of filtering nonwovens.

### 3.5. Antimicrobial Activity—Dynamic Method

The antimicrobial activity of the produced multifunctional polymeric compound WSB_1_ and the reference sample WSB_0_ was evaluated for *Staphylococcus aureus*. The results are shown in Table 6.

The filtration performance of both WSB_0_ and WSB_1_ against an *S. aureus* aerosol was high, amounting to 99.96% and 99.86%, respectively.

Additionally, *S. aureus* counts were performed at time 0 and after 24 h of incubation and the survival index and antimicrobial activity were performed after 24 h. The results are given in Table 7.

*S. aureus* counts during incubation ranged from 1.17 × 10^7^ to 1.55 × 10^7^ CFU/sample, and after 24 h from 8.84 × 10^5^ to 1.11 × 10^7^ CFU/sample. The survival indices (N) were low, amounting to 71.77% for WSB_0_ and 7.55% for WSB_1_. The results revealed that the multifunctional polymeric composite containing a biocide (Biohaloysite) was moderately biocidal and biostatic. The applied *S. aureus* strain was found to be susceptible to the biocide.

### 3.6. Paraffin Oil Mist Penetration and Air Flow Resistance

An evaluation of the basic protective and functional properties (paraffin oil mist penetration and airflow resistance) for the developed multifunctional polymeric composite and the reference nonwoven is summarized in Table 8.

The multifunctional polymeric composite (WSB_1_) designed for filtering respiratory protective devices was characterized by good filtration performance (95%) which corresponds to the protection class FFP2. The applied nonwoven modification technology also decreased paraffin oil mist penetration by around 50%. Due to the incorporation of two additives (SAP and the biocide Biohaloysite variant WSB_1_) air flow resistance through the multifunctional polymeric composite increased compared to the control sample. However, this did not adversely affect the utility parameters; the value obtained is in the protection class FFP1.

## 4. Discussion

Through the innovative method of introducing modifiers into a stream of polymer fibers, with specific technology parameters (Table 3) we obtained a multifunctional nonwoven composite meeting the protective criteria for respiratory protective devices and the functional requirements associated with strenuous work, including adverse microclimate conditions and microbiological hazards.

Two modifiers were simultaneously incorporated into the filtration nonwoven within one technological process using a specially designed melt-blowing die assembly, which led to savings in terms of both energy and raw materials. The process can be used to obtain spatial products with varied properties of their outer surfaces intended for respiratory protective devices. The use of a PP/PET mixture allowed for the improvement of the filtration properties of the nonwoven fabric. The PET admixture improved the nonwoven filtration properties, better filtration efficiency was achieved at lower airflow resistance, and the phenomenon of electrostatic charge fluctuation was reduced. The obtained multifunctional PP/PET/SAP/Biohaloysite melt-blown polymeric composite was characterized by high filtration efficiency and satisfactory airflow resistance at around 200 Pa. In addition, moderate antimicrobial activity was observed for this composite in microbiological tests involving *S. aureus*. In a dynamic test simulating human breathing (inhalation and exhalation), the microorganisms were evenly distributed across samples, which facilitated their exposure to the biocidal agent.

The proposed research methodology, which included infrared spectroscopy and differential scanning calorimetry, allowed for the assessment of the chemical structure of individual variants. The analysis confirmed the presence of the modifiers used in the nonwoven composite (WSB_1_). DSC testing enables the determination of characteristic temperature parameters, thanks to which technological processes can be improved and the manufactured products characterized by better quality and physicochemical properties.

The multifunctional nonwoven composite produced in this work by means of the proposed technology was shown to meet respiratory protective criteria as well as the functional requirements associated with strenuous work while minimizing microbiological hazards and the adverse effects of an unfavorable microclimate. Filtering respiratory devices incorporating the developed composite may be applied under the harsh climatic conditions found in the mining industry, the construction sector, and agriculture.

## Figures and Tables

**Table 1:** Characteristics of the produced filtering nonwovens: WP_0_ was based on polypropylene and WSB_0_ was based on PET-doped polypropylene.

Variant	Conditioning	Parameter	Mean	Standard Deviation	Min.	Max.
WP_0_	No	Paraffin oil mist penetration (%)	15.15	2.01	11.00	19.80
		Airflow resistance (Pa)	174.91	12.08	154.00	204.00
	Yes	Paraffin oil mist penetration (%)	22.83	2.29	19.00	26.40
		Airflow resistance (Pa)	180.85	12.07	159.00	209.00
WSB_0_	No	Paraffin oil mist penetration (%)	10.02	1.17	7.30	12.00
		Airflow resistance (Pa)	129.52	7.17	115.00	145.00
	Yes	Paraffin oil mist penetration (%)	11.38	1.60	8.30	14.00
		Airflow resistance (Pa)	129.95	7.19	15.50	145.50

**Table 2:** Characteristics of the produced multifunctional polymeric composite and the reference nonwoven.

Variant	Parameter	Mean	Standard Deviation	Min.	Max.
WSB_0_	Thickness (mm)	1.73	0.13	1.57	1.91
	Surface density (g/m^2^)	75.81	9.86	58.78	84.59
WSB_1_	Thickness (mm)	2.52	0.39	2.28	3.43
	Surface density (g/m^2^)	136.12	4.51	131.27	145.34

**Table 3:** Technological parameters of the melt-blown process.

Technological Parameter	Value
Temperature of the first extruder zone	280 °C
Temperature of the second extruder zone	270 °C
Air temperature	313 °C
Die assembly temperature	241 °C
Polymer melt temperature	333 °C
Airflow rate	5 m^3^/h
Polymer flow rate	7 g/min

**Table 4:** Microorganisms used in antimicrobial tests.

Microorganisms	Species	Collection Number
Bacteria	*Staphylococcus aureus*	ATCC^1^ 6538

AATC^1^—American Type Culture Collection.

**Table 5:** Water absorption capacity of the filtering nonwovens WSB_0_ and WSB_1_.

Variant		WSB_0_	WSB_1_
Water absorption capacity (g/g)	M^1^	0.59	0.82
	SD^2^	0.14	0.72
	Min.	0.38	0.20
	Max.	0.81	2.44

M^1^—mean, SD^2^—standard deviation.

**Table 6:** Filtration performance of the filtering nonwovens WSB_0_ and WSB_1_ against *S. aureus* aerosol.

Variant	Bacterial Count on Antibacterial Filter, CFU (Colony-Forming Unit)/Sample	Percentage of Bacteria Stopped by the Microbiological Filter	Percentage of Bacteria Stopped by the Tested Nonwoven
WSB_0_	M: 1.26 × 10^5^	0.042	99.96
	SD: 4.92 × 10^4^		
WSB_1_	M: 4.33 × 10^5^	0.140	99.86
	SD: 3.12 × 10^5^		

**Table 7:** *S. aureus* counts on the studied nonwoven variants as well as survival index and microbial activity.

Variant	Bacterial Count on Nonwoven, CFU/Sample		Survival Index N (%)	Biostatic Activity	Biocidal Activity
	t = 0 h	t = 24 h			
WSB_0_	M^1^: 1.55 × 10^7^	M^1^: 1.11 × 10^7^	71.77	-	-
	SD^2^: 1.96 × 10^6^	SD^2^: 4.59 × 10^6^			
WSB_1_	M^1^: 1.17 × 10^5^	M^1^: 8.84 × 10^5^	7.55	1.10	1.24
	SD^2^: 2.15 × 10^4^	SD^2^: 1.86 × 10^5^			

**Table 8:** Characteristics of the basic protective and functional parameters of the tested nonwoven variants.

Variant	Parameter	Mean	Standard Deviation	Min.	Max.
WSB_0_	Paraffin oil mist penetration (%)	10.02	1.17	7.30	12.00
	Airflow resistance (Pa)	129.52	7.17	115.00	145.00
WSB_1_	Paraffin oil mist penetration (%)	5.00	0.89	3.10	6.30
	Airflow resistance (Pa)	199.62	8.86	182.00	219.00
